# Fast immuno-labeling by electrophoretically driven infiltration for intact tissue imaging

**DOI:** 10.1038/srep10640

**Published:** 2015-05-27

**Authors:** Jun Li, Daniel M. Czajkowsky, Xiaowei Li, Zhifeng Shao

**Affiliations:** 1 Shanghai Center for Systems Biomedicine, Shanghai Jiao Tong University, Shanghai 200240, China; 2 School of Biomedical Engineering, Shanghai Jiao Tong University, Shanghai 200240, China; 3 State Key Laboratory for Oncogenes & Related Genes, Shanghai Jiao Tong University, Shanghai 200240, China

## Abstract

Recently developed tissue clearing techniques, where the tissue is embedded within a hydrogel, have revolutionized our ability to resolve fine cellular structures in nearly intact tissues. However, the slow rate of penetration of antibodies within this hydrogel-tissue matrix has become a significantly limiting factor in many experiments, as thick tissues often require weeks to months to be adequately labeled. Increasing the pore size of this matrix has been investigated as a possible solution, but with only modest success. Here, we have systematically examined the diffusional behavior of antibodies and other typically used immuno-labels within this hydrogel-tissue matrix and, surprisingly, found that infiltration occurs at rates similar to those of diffusion in free solution. Therefore, changing the pore size of the matrix would be expected to afford only limited improvement and, instead, some means of active transport is necessary. We show that an electrophoretically-driven approach decreases the delivery time of antibodies by more than 800-fold over simple diffusion, without incurring structural damage. These results, together with the high quality of the images obtained with this method, demonstrate the advantage of this approach, thus significantly broadening the practical range of samples that can now be investigated by whole-mount tissue clearing methods.

Tissue clearing techniques^1^,^2^,^3^,^4^,^5^,^6^,^7^,^8^, especially the recently developed CLARITY^1^, have enabled a nearly complete transformation of intact tissues into optically transparent and macromolecule permeable gel-like assemblies without incurring any changes to their native structure. The power of these approaches has already been demonstrated with the successful three-dimensional imaging of whole-mount organs, such as the brain, kidney, heart, lung, and intestine, using conventional immunofluorescence labeling^9^. The simplicity, convenience, and effectiveness of such methods are widely anticipated to lead to a greater understanding of the intricate relationships between the variety of cells that compose complex tissues and organs^10^,^11^.

However, despite this incredible potential, the widespread application of these approaches has been limited by the notoriously slow penetration of antibodies into the cross-linked tissue. For example, to achieve a uniform labeling of a 5 mm whole-mount mouse brain, more than three weeks is required for each primary and secondary antibody^1^. Hence, when imaging multiple epitopes, these approaches become practically untenable. Since the antibodies move through the samples by simple diffusion, it has been assumed that the pore size of the hydrogel-tissue matrix critically limits antibody infiltration. Studies in which the pore size was changed by varying the cross-linker density have been undertaken, but with only limited improvement in delivery rate^9^.

In this paper, we have used hydrogel-embedded mouse brain sections prepared with CLARITY as a standard specimen and systematically examined the diffusional behavior of immuno-labels of different sizes. Such an understanding enables a prediction of the optimal duration for labeling in a typical experiment. Unexpectedly, we find that the diffusional constants of the labels infiltrating the tissue-hydrogel matrix are no more than four times lower than in free solution. This observation suggests that increasing the pore size of the hydrogel may not, in fact, afford significant improvement in delivery rates. Instead, some means of active transport is required. We thus investigated an electrophoretic approach and found that even at moderate applied electric fields, significantly accelerated delivery rates can be achieved without any change or distortion to the cellular structure. We expect this simple protocol will significantly broaden the range of specimens that can now be practically investigated with intact tissue clearing techniques.

## Results and Discussion

### Measurements of macromolecular diffusion in hydrogel-embedded tissue

**Figure 1:**
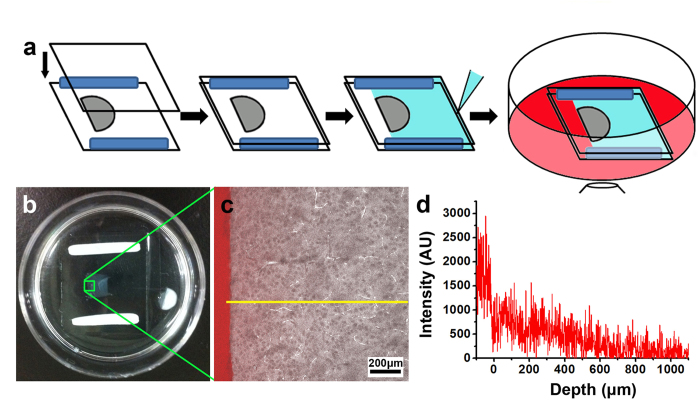
The experimental setup for measuring the antibody diffusion coefficients in the clarified specimen. (**a**) A schematic drawing of the imaging-based assay. (**b**) A photograph of the specimen assembly. (**c**) Fluorescent image of the boxed region in (**b**) 70 min after the addition of fluorescently labeled Fab (red). The image of the tissue is a grey-scale version of the autofluorescence at 488 nm (excitation). (**d**) The fluorescence intensity profile of the red signal in (**c**) along the yellow line.

To monitor the diffusional behavior of various molecules in a cross-linked hydrogel-tissue matrix, we developed an imaging-based strategy in which the one-dimensional diffusional profile of fluorescently labeled molecules from the edge of the specimen is determined with confocal microscopy (Fig. 1a). To ensure that the measurements were obtained from samples similar to those in typical experiments, we investigated 500 μm-thick sections of clarified adult mouse brain prepared following the standard CLARITY protocol (Fig. S1)^12^. To date, four different types of immuno-constructs (namely IgG, F(ab’)_2_, Fab, and nanobody) have been commonly used for immuno-histochemical staining. We thus studied the diffusional properties of fluorescently-labeled versions of these molecules, which range in size from 14 kDa for the nanobody to 150 kDa for IgG. Since the fluorescence intensity is proportional to the molecular concentration, the images reveal the temporal and spatial variation of the molecular concentration in the tissue section, as shown in Fig. 1b–d. To reduce the effect of local variations, an averaged profile was generated by summing the vertical sections of the confocal images (see the Methods section).

**Figure 2:**
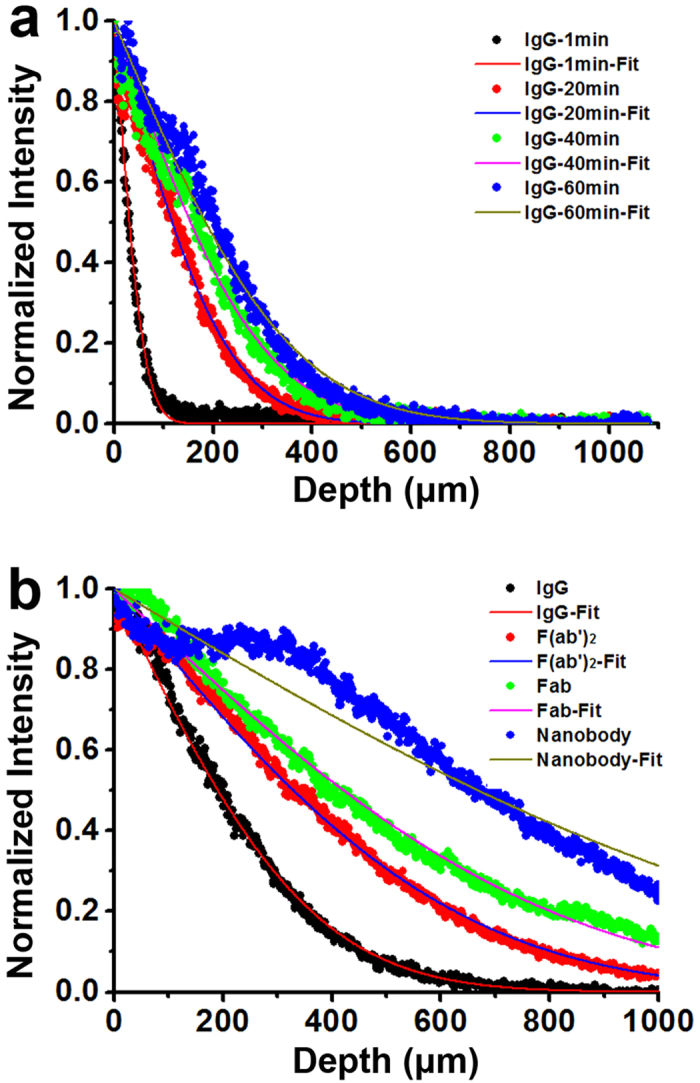
The diffusion profiles of the immuno-labels. (**a**) The measured profiles and fitted curves of IgG molecules at time points of 1, 20, 40 and 60 minutes. (**b**) The diffusion profiles of IgG, F(ab’)_2_, Fab, and nanobody after 60 min.

As shown in Fig. 2a,b, the intensity profiles at all times are well behaved and monotonic. Overall, we found that these curves are well described by Fick’s law of one-dimensional diffusion in a uniform medium^13^, despite local variations in the detailed structure of these sections:
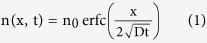
where n(x,t) is the molecular concentration at time t and position x, *erfc* is the complementary error function, n_0_ is the initial concentration, and D is the diffusion coefficient. From these fittings, one can determine the diffusion coefficient, from which the time required to achieve a desired label concentration at a particular tissue depth can be predicted. We note, though, that this measured diffusion coefficient is better described as an “apparent” diffusion coefficient owing to significant structural variations that cause heterogeneous diffusion on a finer scale. Still, this measure should enable at least a useful first-estimate, particularly as there is generally no other *a priori* indicator for this parameter.

Figure 2a shows the fitted diffusion curves of IgG at 1, 20, 40 and 60 minutes and the curves of IgG, F(ab’)_2_, Fab, and the nanobody are plotted after normalization in Fig. 2b. The calculated diffusion coefficients are summarized in Table 1 along with the diffusion coefficients of these molecules measured in free solution (phosphate buffer, pH 7.4)^14^.

As expected, this hydrogel-like matrix does restrict diffusion of the molecules^15^. This is most apparent for the intact IgG, the largest molecule of the immuno-labels, which exhibits a diffusion coefficient of 1.1 × 10^−7^ cm^2^/s, four times lower than that in free solution (4.4 × 10^−7^ cm^2^/s). Hence, while limiting, the difference from free solution in this matrix is only marginal. Thus, it follows that even if the pore size of the matrix were significantly enlarged, the improvement in the labeling time would be moderate at best.

**Figure 3:**
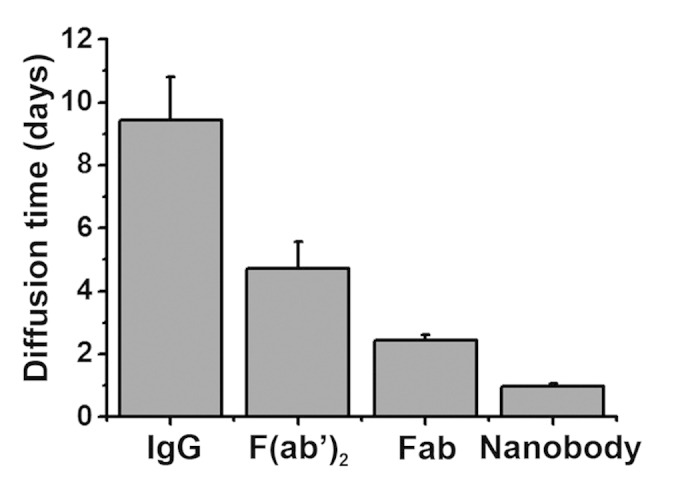
The calculated time (in days) for immuno-labels to completely infiltrate a typical 3 mm-thick hydrogel embedded tissue, based on our measured diffusion coefficients.

The discrepancy between diffusion in this matrix and in free solution further diminishes as the size of the molecule decreases (Table 1). For the newly developed nanobody, the hydrogel-like matrix imposed essentially no restriction on movement, as the measured diffusion coefficient is virtually the same as that expected in free solution (12 × 10^−7^ cm^2^/s and 13 × 10^−7^ cm^2^/s, respectively). Although the diffusion of this nanobody is faster than IgG as expected, the full infiltration of a 3 mm-thick tissue section with this nanobody would still require more than a day (Fig. 3). As nanobodies are the smallest immuno-labels presently available, any further reduction in incubation time would require external forces to accelerate the delivery process. Further, the application of such external forces would be of great advantage if also applicable for intact IgG, for which there exists a more widely available repertoire of useful labels developed against a broad range of epitopes.

### Electrophoretically driven fast immuno-labeling of clarified tissues

We explored whether a simple constant electric field would prove sufficient to enhance the delivery rate of antibodies, taking advantage of their net charge^16^. However, it is also clear that there are likely molecules within the tissue-matrix that also carry net charge, and so it is also possible that local structures sustain stretching or compression under application of this field. Thus, it is important to find proper conditions under which significantly faster diffusion can be achieved without incurring noticeable damage to the local structure.

**Figure 4:**
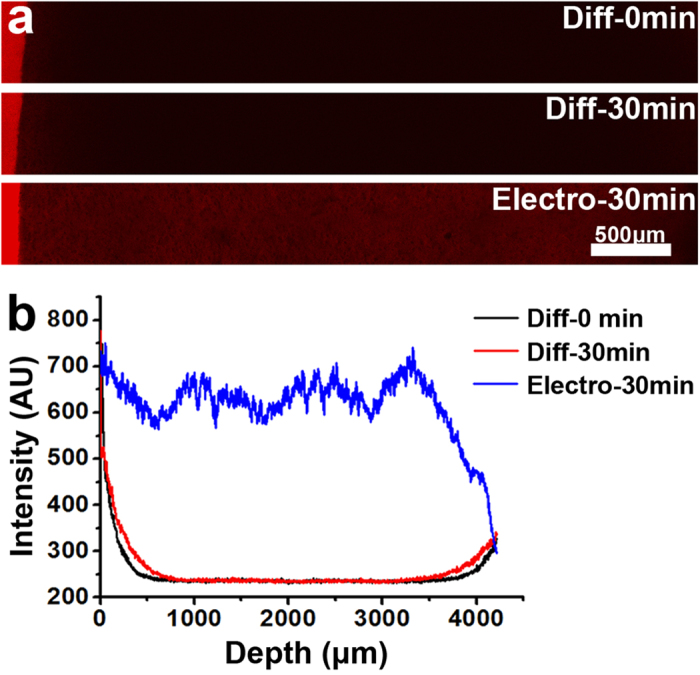
Significantly faster infiltration of IgG driven by an external electric field. (**a**) Fluorescent images of IgG within the clarified tissue at the indicated time points with (Electro) or without (Diff) the application of an external voltage of 25 V across the tissue-matrix. (**b**) The intensity profile of fluorescently labeled IgG molecules delivered by passive diffusion or the external electrical field.

For this purpose, we constructed a chamber in which two platinum wires are used as electrodes to apply a DC electric field across the 500 μm-thick brain section (Fig. S2). As shown in Fig. 4, the improvement in the delivery rate for IgG in these sections is dramatic: in only 30 minutes, the IgG molecules penetrated the entire ~4 mm section at an external voltage of 25 V. Without the external field, the extent of movement owing to passive diffusion of the IgG molecules is negligible. In fact, based on the measured diffusion coefficients (Table 1), the same level of movement by passive diffusion would require ~410 h, about 800-fold longer than that observed under the electric field. With such an improved rate, even multiple rounds of labeling should be achievable in a single day. Although a higher field may further shorten the labeling time, we observed significant increases in temperature beyond a reasonable range with greater electric fields. Based on our experiments, the upper limit of the applied field should be less than 30 V.

**Figure 5:**
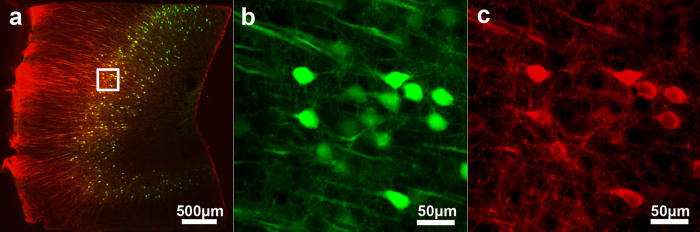
Effective labeling and lack of measurable tissue damage following electrophoretically driven immuno-staining of the mouse brain section. (**a**) Fluorescence image of the immune-labeled Thy1-YFP mouse brain section (green: YFP, red: anti-YFP). (**b**) The endogenous fluorescence (i.e. from YFP) of the boxed region in (**a**). (**c**) Fluorescent image of the anti-YFP label within the boxed region in (**a**).

Using this method, we further examined whether local structures sustain any observable damage under this electric field. To this end, we investigated brain sections from a transgenic mouse in which Thy1-YFP was engineered to express exclusively within the brain^17^. As shown in Fig. 5, the structures observed with standard labeling are virtually indistinguishable from those prepared with the electrophoretically-driven approach (compare with Fig. S1). This demonstrates that with sufficiently low applied voltages, tissue structures retain their original form and local stretching or compression is not significant. Furthermore, signals from the antibody against YFP were highly correlated with those from YFP (Fig. 5). This agreement and the observed uniformity of the antibody signal indicate that our method can indeed be used as an effective approach in place of passive diffusion.

In summary, we have demonstrated a dramatic reduction in the delivery time of immuno-labels within clarified tissues by applying a relatively low external DC field to actively drive the molecules into the tissue. The delivery rates with this method greatly exceeded those obtained by simply using smaller antibody fragments (such as F(ab’)_2_ and Fab) under passive diffusion. Such an electrophoretic approach produced similar labeling results compared with passive diffusion and caused no measurable structural alterations or damage to the tissue. Thus, with this simple improvement, the efficiency and utility of CLARITY for a broader range of intact samples are enhanced, especially when multiple targets are investigated at a higher depth.

## Methods

### Animals

This study was carried out in strict accordance with the guidelines for the care and use of laboratory animals established by the Animal Use and Care Committee of the Shanghai Committee on Animal Care. Animal surgical procedures were approved by the Institutional Animal Care and Use Committee (IACUC) at Shanghai Jiao Tong University, Shanghai, China (Permit Number: SYXK (Shanghai) 2012–0017).

### Clearing the mouse brain with CLARITY

The mouse brains were clarified following the clearing protocol of CLARITY^12^. Briefly, adult mice (6-8 weeks old, stock number: 003782) were anaesthetized and perfused transcardially with the hydrogel monomer solution (HMS), a mixture of 4% (wt) PFA, 4% (wt/vol) acrylamide, 0.05% (wt/vol) bis-acrylamide, 0.25% (wt/vol) VA044, and PBS. Then, the brains were extracted and incubated in the HMS at 4°C for 2 days. The specimen in HMS was degassed to remove residual oxygen using a standard vacuum pump, desiccation chamber and a nitrogen gas supply. The conical tube containing the specimen was kept at 37 °C for 3 hours to polymerize hydrogel monomers. The embedded brain was coronally sectioned on a vibratome (Leica, Germany). Brain sections were then passively cleared using a 4% SDS solution at 37 °C for 2 weeks to completely remove the lipids. The clarified brain sections were then immersed in a 0.5 M sodium borate buffer (pH 8.5) at 37 °C for 24 h to thoroughly remove the remaining SDS.

### Antibody labeling and imaging of the clarified sample

The clarified sections were incubated with antibodies conjugated with the fluorescent dye Dylight650 (1:100, gbdl650, Chromotek) (0.5 M sodium borate, pH 8.5 with 0.1% (wt/vol) Triton X-100) at 37 °C for 5 h followed by washing in the same 0.5 M sodium borate/Triton buffer at 37 °C for 5 h.

Before imaging, the immunolabeled section was further incubated in FocusClear (CelExplorer Labs) for 30 min. Imaging was performed using a Nikon A1Si confocal microscope with a CFI Plan Apo 10 × objective (NA = 0.45, W.D. = 4.0 mm). All image processing was conducted with NIS-Elements (Nikon Instruments Inc., Tokyo, Japan).

### Antibodies used in the passive diffusion and electrophoretic measurements

The IgG (Alexa Fluor 647 goat anti-rabbit IgG (H + L), A21245) was purchased from Life Technologies Corp. (New York, USA). The F(ab’)_2_ (Anti-rabbit IgG (H + L), F(ab’)_2_ Fragment, Alexa Fluor 647 Conjugate, 4414S) was obtained from Cell Signaling Technology Inc. (Massachusetts, USA). The Fab was produced from the above IgG using the Pierce Fab Micro Preparation Kit (44685, Thermo Scientific). The nanobody (GFP-Booster_DyLight650, gbdl650) was purchased from Chromotek GmbH (Planegg-Martinsried, Germany). The final concentration of all molecules was 36 μM.

### Measurement of passive diffusion profiles

A 500-μm thick clarified brain section was placed between two coverslips using BluTack putty clay as a spacer. This assembly was fixed in a 35 mm petri dish using cyanoacrylate glue. Mountgel was used to seal three sides of the section assembly to ensure that molecules can only diffuse in the tissue in one direction. Confocal images were acquired every 10 min.

The recorded fluorescence images were processed by subtracting the background, followed by vertical summation of the signals to generate a one dimensional diffusion curve. The normalized diffusion curves were fitted to Equation (1) and the “apparent” diffusion coefficient was obtained.

### External electrical field driven antibody delivery and immuno-labeling

The brain section assembly was prepared following the same procedure as that used for passive diffusion measurements, except for the Mountgel sealing. Two platinum electrodes (267201-2G, Sigma) were placed on each side of the section with a separation distance of about 2.2 cm from each other. The antibody delivery process with or without an external electrical field was monitored in real time by confocal microscopy. Image processing was the same as that presented above. Immuno-labeling of the transgenic mouse brain sections was performed using an anti-GFP antibody (1:333 dilution in 0.1 M sodium borate (pH 8.5), A31852, GFP Rabbit IgG Antibody Fraction, Alexa Fluor 647 Conjugate, Life Technologies Corp), by first applying a voltage of 25 V for 30 min, followed by an incubation period of 1.5 h, then washing the sample with 0.1 M sodium borate (pH 8.5), and finally applying a voltage of 25 V for 30 min to drive the free antibodies from the tissue.

## Additional Information

**How to cite this article**: Li, J. *et al*. Fast immuno-labeling by electrophoretically driven infiltration for intact tissue imaging. *Sci. Rep.* **5**, 10640; doi: 10.1038/srep10640 (2015).

## Supplementary Material

Supporting Information

## Figures and Tables

**Table 1:**
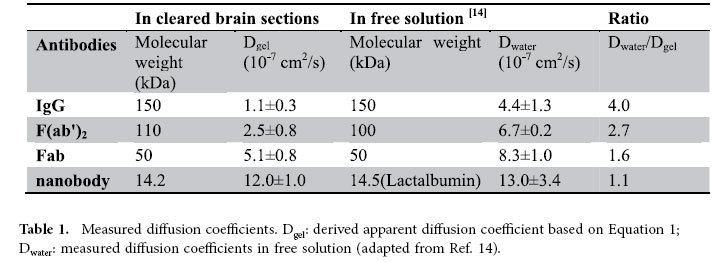
Measured diffusion coefficients.
