# Supplementary material for: Fast immuno-labeling by electrophoretically driven infiltration for intact tissue imaging
Source: Sci Rep. 2015 May 27;5:10640. doi: 10.1038/srep10640 (PMC4603706; doi:10.1038/srep10640)
Supplement: Supporting Information [file srep10640-s1.pdf]

**Supporting Information**

# Fast immuno-labeling by electrophoretically driven infiltration for intact tissue imaging

*Jun Li*[1], *Daniel M. Czajkowsky*[2], *Xiaowei Li*[2*], *Zhifeng Shao*[2,3*]

[1]Shanghai Center for Systems Biomedicine, Shanghai Jiao Tong University, Shanghai 200240, China.

[2]School of Biomedical Engineering, Shanghai Jiao Tong University, Shanghai 200240, China.

[3]State Key Laboratory for Oncogenes & Related Genes, Shanghai Jiao Tong University, Shanghai 200240, China.

Email: xl3a@sjtu.edu.cn; zfshao@sjtu.edu.cn

**Supplementary figures**

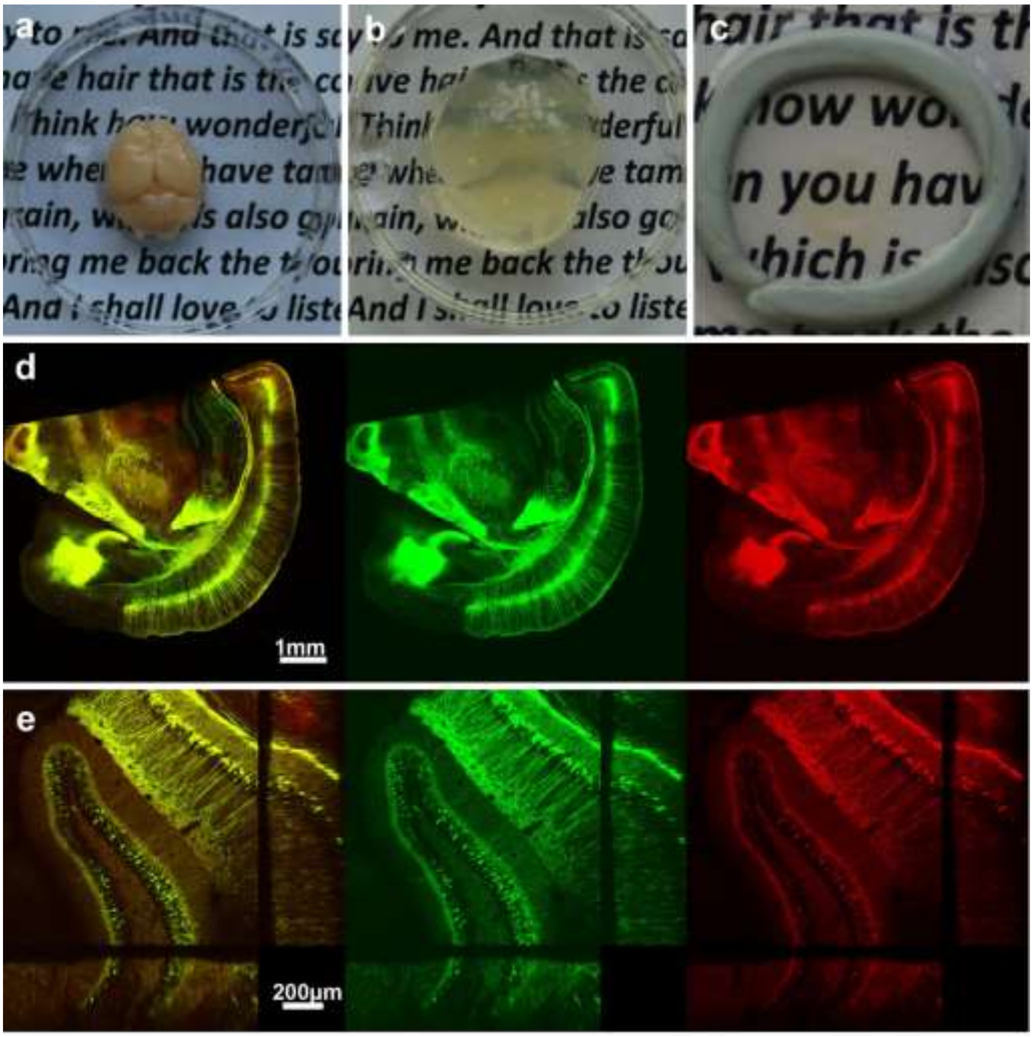


Figure S1. Images of clarified adult mouse brain. (a) A mouse brain before CLARITY. (b) The same mouse brain after CLARITY. (c) A 500-μm thick section of the clarified mouse brain immersed in FocusClear. (d) Fluorescent images of a brain section from a mouse expressing Thy1-YFP obtained with confocal microscopy (green: GFP, red: anti-GFP). (e) Higher magnified images of the Thy1-YFP mouse brain section shown in (d). In each panel, the main image is the x-y cross-section, the image on the right is the y-z cross section, and the image at the bottom is the x-z cross-section.

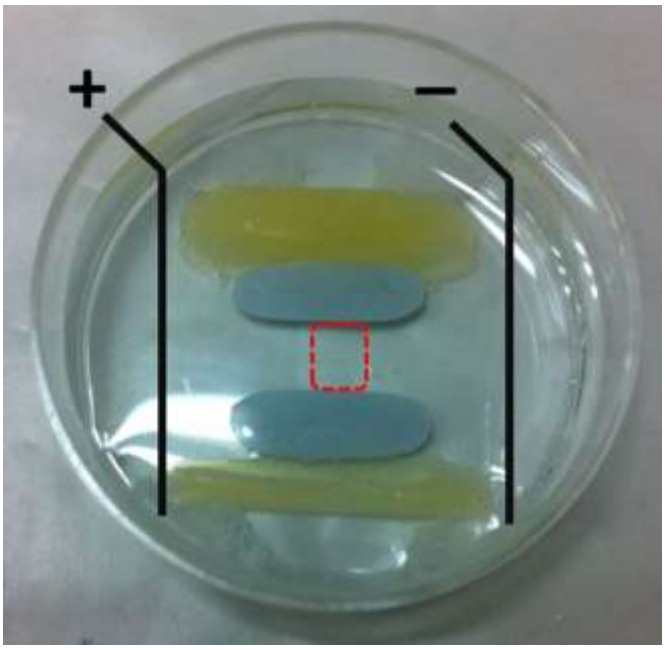


Figure S2. Schematic diagram of the sample assembly used to electrophoretically drive the antibodies within the clarified tissue. Superimposed on a photograph of the assembly is a red box indicating the location of the brain section and black lines representing the two platinum electrodes.
